# Vitamin K antagonist anticoagulant usage is associated with increased incidence and progression of osteoarthritis

**DOI:** 10.1136/annrheumdis-2020-219483

**Published:** 2021-03-03

**Authors:** Cindy G Boer, Ingrid Szilagyi, N Long Nguyen, Tuhina Neogi, Ingrid Meulenbelt, M Arfan Ikram, André G Uitterlinden, Sita Bierma-Zeinstra, Bruno H Stricker, Joyce B van Meurs

**Affiliations:** 1 Department of Internal Medicine, Erasmus MC, University Medical Center, Rotterdam, The Netherlands; 2 Department of General Practice, Erasmus MC, University Medical Center, Rotterdam, The Netherlands; 3 Section of Rheumatology, Department of Medicine, Boston University Medical Campus, Boston, Massachusetts, USA; 4 Section Molecular Epidemiology, Department Biomedical Data Sciences, Leiden University Medical Center, Leiden, The Netherlands; 5 Department of Epidemiology, Erasmus MC, University Medical Center, Rotterdam, The Netherlands

**Keywords:** osteoarthritis, epidemiology, pharmacogenetics

## Abstract

**Objectives:**

Vitamin K is hypothesised to play a role in osteoarthritis (OA) pathogenesis through effects on vitamin K-dependent bone and cartilage proteins, and therefore may represent a modifiable risk factor. A genetic variant in a vitamin K-dependent protein that is an essential inhibitor for cartilage calcification, matrix Gla protein (MGP), was associated with an increased risk for OA. Vitamin K antagonist anticoagulants (VKAs), such as warfarin and acenocoumarol, act as anticoagulants through inhibition of vitamin K-dependent blood coagulation proteins. VKAs likely also affect the functioning of other vitamin K-dependent proteins such as MGP.

**Methods:**

We investigated the effect of acenocoumarol usage on progression and incidence of radiographic OA in 3494 participants of the Rotterdam Study cohort. We also examined the effect of *MGP* and *VKORC1* single nucleotide variants on this association.

**Results:**

Acenocoumarol usage was associated with an increased risk of OA incidence and progression (OR=2.50, 95% CI=1.94–3.20), both for knee (OR=2.34, 95% CI=1.67–3.22) and hip OA (OR=2.74, 95% CI=1.82–4.11). Among acenocoumarol users, carriers of the high *VKORC1(BB*) expression haplotype together with the *MGP* OA risk allele (rs1800801-T) had an increased risk of OA incidence and progression (OR=4.18, 95% CI=2.69–6.50), while this relationship was not present in non-users of that group (OR=1.01, 95% CI=0.78–1.33).

**Conclusions:**

These findings support the importance of vitamin K and vitamin K-dependent proteins, as MGP, in the pathogenesis of OA. Additionally, these results may have direct implications for the clinical prevention of OA, supporting the consideration of direct oral anticoagulants in favour of VKAs.

**Key messages**

**What is already known about this subject?**

- Osteoarthritis (OA) is the most common form of arthritis worldwide, affecting 320 million people, and is a leading cause of disability. To date, there are no disease-modifying therapies available, and treatment development has been hampered by existence of only few recognised modifiable risk factors.
- Vitamin K and vitamin K-dependent proteins, such as matrix Gla protein (MGP), have been implicated in OA by epidemiological studies, genetic studies and subsequent in functional genomics studies, indicating vitamin K as possible modifiable risk factor for OA.

**What does this study add?**

- This study shows that the use of vitamin K antagonist anticoagulants (VKAs) significantly increases the risk of progression of hip and knee OA, by inhibiting the vitamin K pathway.
- This study also demonstrates that known OA genetic risk variants in *MGP* and pharmacogenetic variants known to affect vitamin K metabolism increase the risk of OA progression when using VKAs.

**How might this impact on clinical practice or future developments?**

- The findings suggest the consideration of novel (or direct) oral anticoagulants in favour of VKAs, such as acenocoumarol and warfarin, in people with OA.

## Introduction

Osteoarthritis (OA) is a chronic disabling joint disease that also increases in prevalence with age. It is the most common form of arthritis, one of the fastest growing chronic diseases worldwide,^1^ and is the fourth leading cause of years lived with disability globally.^2^ To date, there are no known therapies that can alter its progression or prevent its occurrence. Apart from obesity and knee injury, very few other modifiable risk factors have been identified. Vitamin K has been hypothesised to play a role in OA pathogenesis through its effects on several vitamin K-dependent bone and cartilage proteins,^3^ and therefore may represent a modifiable risk factor. A number of observational studies reported an association between vitamin K status and prevalence and incidence of OA.^4–6^ There has been one modestly sized clinical trial studying the effect of vitamin K supplementation on OA progression. This ancillary study, originally designed to study vascular calcification, reported no overall beneficial effects of vitamin K supplementation. However, in individuals with insufficient vitamin K levels at baseline, a beneficial effect was observed.^7^ No studies to date have evaluated the relation between vitamin K antagonist anticoagulants (VKAs) and OA, which can be expected to result in low vitamin K functioning, which may lead to increased OA incidence or progression.^4–6^

Vitamin K is an essential cofactor in the post-translational γ‐carboxylation of glutamic acid to form γ‐carboxyglutamic acid (Gla) residues, which confer functionality to Gla proteins. VKAs deplete the active form of vitamin K by inhibiting the enzyme vitamin K epoxide reductase complex 1 (*VKORC1*). Genetic variants of *VKORC1* account for approximately 25% of the variance in VKA dose.^8^ Matrix Gla protein (*MGP*) is a vitamin K-dependent Gla protein that is an essential inhibitor of cartilage and vascular mineralisation.^9 10^ Recently, genome-wide association study (GWAS) and functional studies identified *MGP* to be causally involved in OA.^11 12^

VKAs such as warfarin and acenocoumarol are primarily prescribed for the prevention of thromboembolic events in patients with atrial fibrillation (AF).^13^ With ageing-related increases in prevalence of AF, the projected number of individuals with AF needing anticoagulation is predicted to rise to 17.9 million by 2060 in the European Union.^14^ While a new class of anticoagulants are available, the non-vitamin K oral anticoagulants (NOACs), VKAs are still widely prescribed, particularly to older adults.^15^ Whether long-term VKA use with resultant impairment of vitamin K-dependent proteins such as *MGP* increases risk of OA incidence or progression is not known. Given the high prevalence of VKA users in addition to the high prevalence of OA globally, clarifying this relationship would have substantial public health impact by identifying a potentially modifiable risk factor for OA.

We therefore examined the relation of VKA use to progression and incidence of hip and knee OA in two subcohorts of the large prospective population-based cohort of the Rotterdam study (RS). We additionally examined how the impact of VKA use varies by the presence of the *MGP* risk allele that influences *MGP* expression and single nucleotide variations (SNVs) affecting *VKORC1* gene expression, which impact VKA dosage.

## Methods

### Study population and clinical data

The Rotterdam Study (RS) is a large prospective population-based cohort study ongoing since 1990 to study determinants of chronic disabling diseases in the elderly.^16^ It consists of separate subcohorts (RS-I, RS-II, RS-III). All RS cohort participants live in the Ommoord district of the city of Rotterdam, the Netherlands. Residents of 55 years and older were first recruited in 1990. In 2000, a second cohort, RS-II, was started with individuals who had become 55 years of age or moved into the study district since the start of the study. Follow-up data were collected at follow-up visits every ~5–6 years. Details of the design and rationale of the RS have been published elsewhere.^16^ Participant measurements at baseline and follow-up were obtained during visits to the research centre for physical examinations, computerised pharmacy records and from home interviews. Our study included participants of RS-I and RS-II for whom radiographs of knee and hip joints at baseline and follow-up visit were present, obtained and scored (online supplemental figure S1). Additional information included sex, age at baseline visit, body mass index (BMI, kg/m^2^), physical activity (metabolic equivalent of task/hours per week), smoking (never, former and current smoker), locomotor disability, education level (UNESCO education classification), diabetes mellitus, hypertension, femoral neck bone mineral density (FN-BMD), HDL/total cholesterol ratio and the Stanford Health Assessment Questionnaire (see online supplemental text for details).

10.1136/annrheumdis-2020-219483.supp1 Supplementary data

10.1136/annrheumdis-2020-219483.supp2 Supplementary data

The RS has been approved by the institutional review board (Medical Ethics Committee) of the Erasmus Medical Center and by the review board of The Netherlands Ministry of Health, Welfare and Sports. The approval has been renewed every 5 years (MEC 02.1015). All participants provided written informed consent for participation in the RS.

### Incidence and progression of OA

Our study included participants of RS-I and RS-II for whom radiographs of knee and hip joints at baseline and follow-up visit were obtained and scored by trained medical professionals for OA severity using Kellgren and Lawrence Grade (KLG)^17 18^ (online supplemental figure S1). Individuals who had at baseline locomotor disability were excluded from our study population^19^ (online supplemental figure S1). We analysed OA incidence and progression together, as both definitions cannot be accurately defined based on radiographic examination alone, and by combining both into one definition reduces this bias.^20^ We evaluated any OA progression, defined as an increase of KLG between baseline and follow-up of ≥1 and/or joint replacement; if baseline KLG was 0, progression was defined as an increase of KLG ≥2 (incidence).^21^ Joints with a baseline KLG of 4 or baseline joint replacement were excluded from analysis (online supplemental figure S1). OA progression was defined in a joint-specific and side-specific manner (knee, hip; left and right). Joints with no progression of OA comprised the referent group. Joints with missing data were excluded (online supplemental figure S1), with the exception of joints with missing baseline data and a KLG of ≤1 at follow-up, which were included in the referent group (online supplemental figure S1).

### Vitamin K antagonist anticoagulants

For each participant, we extracted the usage of VKAs (acenocoumarol) for the period between baseline visit (RS-I-1, RS-II-1) and follow-up visits (RS-I-3, RS-II-2), from computerised pharmacy data (online supplemental text). Acenocoumarol is the main prescribed VKA in the Netherlands as warfarin is not registered for use as a drug. All participants taking VKAs attended an anticoagulation clinic, which is standard practice in the Netherlands.^22^ We excluded participants who were taking VKA (n=148) during the baseline visit to avoid prevalent user bias. We defined VKA usage as any acenocoumarol usage during the period between the baseline (RS-I, RS-II) and follow-up visit (RS-I-3, RS-II-2), regardless of duration or dosage. To examine the effects of increasing duration of use, we defined duration of use by tertiles: ≤180 days, between >180 days and ≤556 days of use, and >556 days of use.

### Genetic data and haplotype analysis

Methods for DNA isolation, genotyping, quality control and data processing have been described elsewhere.^11^ Data from 11 SNVs were extracted from the genotyped and imputed genetic dataset: the *MGP* SNV previously found associated with OA^11^ and 10 SNVs needed for the *VKORC1* H-haplotypes as described on the PharmGKB database^23^ (online supplemental table S1). Haplotypes were inferred from all available genotypes (N=8448), using imputed genotype dosage data (HRC panel v.1.1^24^) and the R-package haplo.stats.^25^ Haplotypes were grouped based on VKA maintenance dose/*VKORC1* expression association: (A) low-dose VKA requirement/low *VKORC1* expression and (B) high-dose VKA requirement/high *VKORC1* expression.^26^ Study participants were further stratified into: low expression/dose (AA), intermediate expression/dose (AB) and high expression/dose (BB) groups (table 1 and online supplemental table S1).

10.1136/annrheumdis-2020-219483.supp3 Supplementary data

**Table 1:** Study population characteristics

	Study population (RSI and RSII)	
	Non-users (N=3255)	Acenocoumarol users (N=239)
*General characteristics*		
RSI	2394 (73.5%)	207 (86.6%)
RSII	861 (26.5%)	33 (13.4%)
Age, years	64.2 (6.3)	66.6 (6.8)
Females (%)	1778 (54.6%)	117 (49.0%)
Follow-up period, months, median (IQR)	76.1 (55.2, 78.5)	76.7 (75.1, 79.4)
Smoking status		
Never	1028 (31.6%)	65 (27.2%)
Former smoker	1538 (47.3%)	119 (49.8%)
Current smoker	689 (21.2%)	55 (23.0%)
Education		
Primary education	385 (11.7%)	34 (14.2%)
Intermediate general education	1433 (44.0%)	103 (43.1%)
Higher general education	1026 (31.5%))	69 (28.9%)
Higher vocational education/University	411 (12.6%)	33 (13.8%)
Physical activity (MET/week)	89.9 (44.69)	78.27 (41.5)
Body mass index (BMI), kg/m^2^	26.3 (3.5)	26.9 (3.5)
Total cholesterol/HDL ratio	5.0 (1.6)	5.3 (1.5)
Hypertension (%)	1709 (52.3%)	143 (59.8%)
Diabetes mellitus (%)	386 (11.9%)	41 (17.2%)
Femoral neck BMD (g/cm^2^)	0.88 (0.13)	0.88 (0.12)
Osteoarthritis status*		
Hip OA (%)	65 (2.0%)	10 (4.6%)
Knee OA (%)	262 (8.5%)	26 (12.0%)
*MGP risk allele (rs1800801*)		
Non-risk allele (A/A)	1168 (38.6%)	79 (36.3%)
Risk allele carrier (T/*)	1856 (61.4%)	137 (63.4%)
*VKORC1* Haplotype groups†		
Low—AA	447 (14.8%)	42 (19.5%)
Intermediate—AB	1425 (47.3%)	92 (42.8%)
High—BB	1142 (37.9%)	81 (37.7%)
*MGP* risk allele carrier and *VKORC1* BB-haplotype	684 (22.7%)	51 (23.7%)

*Osteoarthritis at baseline was defined as radiographic OA, Kellgren-Lawrence score ≥2 in either the left or right or both investigated joints.

†*VKORC1* groups based on *VKORC1* H-Haplotypes and their association with *VKORC1* expression/VKAs maintenance dosage; low: low *VKORC1* expression and associated with lower required dosage; high: high *VKORC1* expression and associated with higher required dosage, also see online supplemental table S1.

BMD, bone mineral density; HDL, high-density lipoprotein; MET, metabolic equivalent of task hours; OA, osteoarthritis; RS, Rotterdam Study.

### Statistical analysis

We evaluated the relation of VKA to the risk of overall progression of OA of either the knee or hip using logistic regression with generalised estimating equations (GEE) to account for correlations between joints within an individual.^21^ We repeated the analyses stratified by *VKORC1* and *MGP* genotype/haplotype. The following covariates were included in all analyses: sex, age, BMI, physical activity, smoking, education level, diabetes mellitus, hypertension, FN-BMD, HDL/total cholesterol ratio, baseline OA severity, time between follow-up visits, joint modelled and RS cohort (online supplemental text).

## Results

### Relation of acenocoumarol use to OA progression

A total 3494 of participants of two large prospective older-age population-based cohorts, the Rotterdam Study (RS), were included in this study, with RS-I contributing 2601 individuals, while RSII contributed 894 participants.^16^ See table 1 for the general characteristics of the study population. At baseline, there were 363 individuals with OA (KLG ≥2), 75 with hip OA and 288 with knee OA. We identified 239 new users of acenocoumarol (VKA) (RS-I n=207, RS-II n=33) in our study population in our follow-up period.

When we examined the incidence/progression of OA in acenocoumarol users and non-users, there was a >2-fold higher risk for overall OA incidence/progression (ie, OA of the knee or hip) in acenocoumarol users compared with non-users (OR=2.50, 95% CI=1.94–3.20; table 2, online supplemental table S2). This association was also observed in each subcohort (RS-I and RS-II) separately (online supplemental table S3) and for OA incidence and OA progression separately (online supplemental table S4). Overall OA incidence/progression risk estimates remained similar for longer duration of acenocoumarol use (tertiles): ≤180 days (OR=2.82, 95% CI=1.90–4.20), >180 days and ≤556 days (OR=2.94, 95% CI=2.00–4.32), with only a reduction of risk with long-term use, >556 days of acenocoumarol use (OR=1.74, 95% CI=1.10–2.76) (table 2).

**Table 2:** Association between acenocoumarol use and risk of OA incidence and progression in RSI and RSII

	Overall osteoarthritis progression					Overall progression of knee osteoarthritis					Overall progression of hip osteoarthritis					
	Joints N*	Incidence/Progression N (%)	OR adj.†	95% CI	P value	Joints N*	Incidence/Progression N (%)	OR adj.†	95% CI	P value		Joints N*	Incidence/Progression N (%)	OR adj.†	95% CI	P value
Non-users	12 594	506 (4.0%)	1	–	–	6162	329 (5.3%)	1				6432	177 (2.8%)	1	–	–
Users	863	94 (10.9%)	2.5	1.94 to 3.20	>0.001	426	55 (12.9%)	2.34	1.69 to 3.22	>0.001		437	39 (8.9%)	2.74	1.82 to 4.11	>0.001
Duration of acenocoumarol usage																
*Non users*	12 594	506 (4.0%)	1	–	–	6162	329 (5.3%)	1	–	–		6432	177 (2.8%)	1	–	–
≤180 days	279	35 (12.5%)	2.82	1.90 to 4.20	>0.001	144	15 (10.4%)	1.78	1.01 to 3.18	4.8×10^–02^		135	20 (14.8%)	4.84	2.73 to 8.56	>0.001
>180 days and ≤556 days	285	36 (12.6%)	2.94	2.00 to 4.32	>0.001	135	20 (14.8%)	2.69	1.62 to 4.46	>0.001		150	16 (10.7%)	3.33	1.79 to 6.18	>0.001
*>556* days	299	23 (7.7%)	1.74	1.10 to 2.76	1.9×10^–02^	147	20 (13.6%)	2.65	1.57 to 4.46	>0.001		152	3 (2.0%)	0.27	0.18 to 1.85	0.35

Incidence and progression of osteoarthritis (OA) in RS-I and RS-II within the follow-up time associated with acenocoumarol use. Model used is a GEE (generalised estimated equations) multivariate logistic regression model including acenocoumarol use and adjusted for age, sex, BMI, smoking, time between baseline and follow-up visit, baseline OA severity in Kellgren-Lawrence score, joint modelled, femoral neck BMD, HDL/total cholesterol ratio, physical activity, education level, hypertension, diabetes mellitus and Rotterdam Study cohort.

Progression: number of joints showing overall progression of either hip or knee joints or both. Acenocoumarol usage examined by tertiles: first: ≤180 days, second: >180 and ≤556 days, third: >556 days of acenocoumarol use.

*Number of individual knee and/or hip joints studied from RSI and RSII (online supplemental figure S1 for exclusions).

†Unadjusted (raw) ORs are reported in online supplemental table S2.

BMI, body mass index; HDL, high-density lipoprotein; RS, Rotterdam Study.

Increased risk of overall OA incidence/progression in acenocoumarol users was also observed in the knee and hip joints separately (table 2) (knee OA (OR=2.34, 95% CI=1.69–3.22) and hip OA (OR=2.74, 95% CI=1.82–4.11)), as well as in each subcohort separately (online supplemental table S3). Interestingly, longer duration of acenocoumarol use does seem to have slight different effects on overall knee OA incidence/progression than on hip OA. Knee OA risk seems to increase for longer duration of acenocoumarol use, whereas risk for hip OA seems to decrease for longer durations of acenocoumarol use. These differences may represent true biological differences between the joints. However, this is more likely the effect of low statistical power (tertiles analysis), the statistical power difference between the joints as there were more cases of overall knee OA incidence/progression (n=385 joints) in our study cohorts than for hip OA (n=216 joints). In addition, the absence of a stronger effect in long-term acenocoumarol users could be explained by depletion of susceptible bias.^27^ This bias suggests that the cohort is depleted of all its susceptible subjects who had the event, OA incidence/progression, early on. Seemingly decreasing the risk with longer acenocoumarol (exposure) use. Thus, the remaining acenocoumarol users will include fewer OA incidence/progression predisposed subjects over the longer follow‐up, causing the risk to decrease over time.

### MGP, VKORC1 genetics and acenocoumarol affect OA progression

Maintenance dosages of VKAs are dependent on genetic variants (SNVs) affecting *VKORC1* expression activity.^22 28^ This altered *VKORC1* expression can also affect MGP γ-carboxylation, as *VKORC1* is needed for γ-carboxylation by vitamin K, a process that is needed to activate MGP. We therefore examined the extent to which acenocoumarol users with a genetic predisposition for decreased *MGP* and/or altered *VKORC1* expression had an altered risk for overall incidence/progression of OA. The study population was stratified in *MGP* risk allele carriers (T/*) and non-carriers (A/A) (table 1).

Acenocoumarol users among both *MGP* genotype groups had a significantly higher risk of overall OA incidence/progression than non-users (table 3). Using the *VKORC1*-Haplotypes, the study population could be also stratified into low (AA), intermediate (AB) and high (BB) *VKORC1* expression/VKA dose groups^26^ (table 1 and online supplemental table S1). Similar to the *MGP* genotypes, we observed no effect of the VCORC1 genotypes on the risk for overall OA incidence/progression (table 3), although the *VKORC1-*BB group had the highest risk of overall OA incidence/progression (OR=3.35, 95% CI=2.22–5.05, table 3).

**Table 3:** Acenocoumarol use interacts with *MGP* OA risk variants and *VKORC1* haplotype groups, leading to increased risk of overall incidence/progression of osteoarthritis

Acenocoumarol use	Joints*	Incidence/Progression	OR	95% CI	OR	95% CI	P value
	N	N (%)			Adj.	Adj.	Adj.
*MGP rs1800801 alleles*							
Non-users MGP non-risk allele carriers (A/A)	4332	205 (4.7%)	1	–	1	–	–
Non-users MGP risk allele carriers (T/*)	7211	274 (3.8%)	0.83	0.69 to 1.00	0.86	0.71 to 1.04	0.11
Users MGP non-risk allele carriers (A/A)	286	26 (9.1%)	2.11	1.37 to 3.24	2.01	1.29 to 3.13	2.0×10^–03^
Users MGP risk allele carriers (T/*)	492	58 (11.8%)	2.82	2.08 to 3.84	2.57	1.87 to 3.54	1.1×10^–08^
*VKORC1 haplotype groups*							
Non-users low VKORC1 group (AA)	1735	75 (4.3%)	1		1	–	–
Non-users intermediate VKORC1 gGroup (AB)	5525	203 (3.7%)	0.84	0.64 to 1.11	0.84	0.64 to 1.10	0.2
Non-users high VKORC1 group (BB)	4448	201 (4.5%)	1.05	0.80 to 1.37	1.04	0.79 to 1.36	0.8
Users low VKORC1 group (AA)	152	16 (10.1%)	2.6	1.48 to 4.59	2.31	1.28 to 4.17	5.3×10^–03^
Users intermediate VKORC1 group (AB)	317	24 (7.6%)	1.81	1.13 to 2.92	1.53	0.34 to 2.51	9.1×10^–02^
Users high VKORC1 group (BB)	305	42 (13.8%)	3.53	2.37 to 5.27	3.35	2.22 to 5.05	7.2×10^–09^

Overall progression of osteoarthritis (OA) in RSI and RSII within the follow-up time associated with acenocoumarol use effect *MGP* rs1800801 OA risk variant and *VKORC1* expression/VKA dosage haplotypes. Model used is a GEE (generalised estimated equations) multivariate logistic regression model including acenocoumarol use adjusted (Adj.) for age, sex, BMI, smoking, time between baseline and follow-up visit, baseline OA severity in Kellgren-Lawrence score, joint modelled, femoral neck BMD, HDL/total cholesterol ratio, physical activity, education level, hypertension, diabetes mellitus and Rotterdam Study cohort. *VKORC1* haplotype groups are based on the H haplotypes, see online supplemental table S1. For *MGP* risk variants and carriers, see Table 1. Progression: number of joints showing overall OA progression; T/*: MGP osteoarthritis risk variant carrier (T/A) or (T/T).

*Number of individual knee and hip joints included in the analysis.

BMD, bone mineral density; BMI, body mass index.

**Figure 1:**
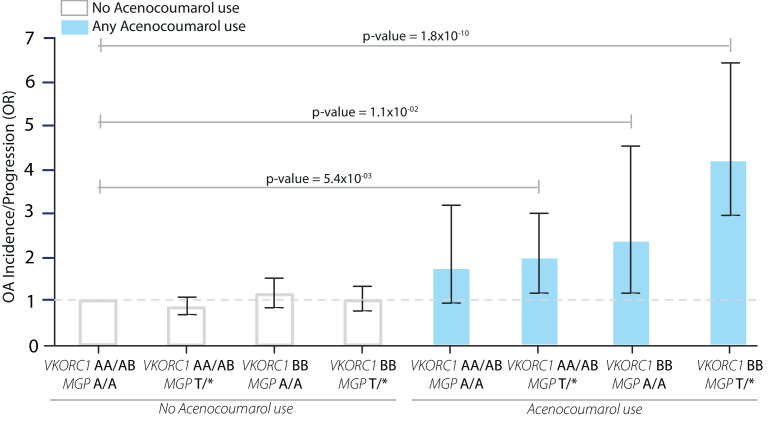
Acenocoumarol use interacts with *MGP* osteoarthritis (OA) risk single nucleotide variants (SNVs) and *VKORC1* haplotype groups, leading to increased risk of overall progression of OA. *VKORC1* haplotype groups are based on the *VKORC1* H-haplotypes, which can be divided into three groups based on their VKA dosage/*VKORC1* expression association: AA=low VKA dose/*VKORC1* expression; AB=intermediate VKA dose/*VKORC1* expression; and BB=high VKA dose/*VKORC1* expression. Acenocoumarol use in *VKORC1* BB haplotype carriers and *MGP* risk allele carriers and OA risk. OR, CI and p value based on GEE multivariate logistic regression adjusted for age, sex, BMI, smoking, time between baseline and follow-up visit, baseline OA severity in Kellgren-Lawrence score, joint modelled, femoral neck BMD, HDL/total cholesterol ratio, physical activity, education level, hypertension and diabetes mellitus. A/A: non-carrier of *MGP* risk allele; T/*: carrier of *MGP* risk allele; *VKORC1 AA*: homozygous *VKORC1 A* haplotype carriers; *VKORC1 BB*: homozygous *VKORC1 BB* haplotype carriers. error bars indicate 95% CI for the OR. P values belong to depicted OR. See online supplemental table S3 for the exact values depicted in this graph.

As individuals can be both carriers of *MGP* risk alleles and *VKROC1* haplotypes, we examined the combined effects of the *VKORC1-BB* haplotype and *MGP* risk allele carriers (T/*). We stratified our study population into *VKORC1* BB-haplotype or AA/AB carriers, which was then further stratified into carriers (T/*) and non-carriers (A/A) of the *MGP* risk allele, rs1800801 (figure 1 and online supplemental table S5). Acenocoumarol users whom either were carriers of the *MGP* risk alleles (T/*) or carriers of the *VKORC1 BB-*haplotype had a significant increased risk of overall OA incidence/progression. Individuals whom were carriers of both the *VKORC1 BB-haplotype* and the *MGP* risk allele had a fourfold increased risk of OA incidence/progression (OR=4.18, 95% CI=2.69–6.50, figure 1). Interestingly, *VKORC1* AA/AB-haplotype carriers who were not carriers of the MGP risk allele (A/A) did not have a significant increased risk of overall OA progression/incidence when using acenocoumarol (OR=1.72, 95% CI=0.93–3.19, figure 1).

## Discussion

We demonstrated that use of the acenocoumarol was associated with a higher risk of overall OA incidence/progression in non-users. We observed that the increased OA risk in acenocoumarol users varied based on genetic variants affecting the vitamin K cycle with VKA use and *MGP* risk allele status. Acenocoumarol users with the *MGP* risk allele and *VKORC1* BB-haplotype had a fourfold higher risk for overall OA incidence/progression.

The *VKORC1* BB-haplotype is associated with a higher expression of *VKORC1*, associated with greater vitamin K activity; however, individuals who are *VKORC1* BB-haplotype carriers also require and receive higher dosages of VKA for the desired anticoagulation effect.^26^ Intuitively, the anticoagulation, amount of VKORC1 inhibition, should be similar in all users regardless of *VKORC1* haplotype since the amount of anticoagulation is the dosing measurement, not *VKORC1* expression. Previous research in animal models has indicated that vitamin K availability levels differ significantly between tissues^29 30^ and warfarin affects vitamin K inhibition differently in liver compared with bone.^31–33^ In liver, another enzyme in addition to *VKORC1* is available for the recycling of vitamin K into its active form, *NQO1* (*NAD(P)H quinone oxidoreductase 1*). This enzyme is not present in bone tissue, causing the bone tissue to be potentially more susceptible to VKA dosages than liver tissue.^32–34^ Thus, we propose the following hypothesis: that the higher VKA dosages in *VKORC1* BB-haplotype carriers, needed for desired inhibition of vitamin K-dependent blood coagulation proteins in the liver, might be too high of a dosage for *VKORC1* functioning in the joint. This hypothesis, however, needs to be further examined in functional studies, particularly in human functional studies.

Oral VKAs, which include acenocoumarol and warfarin, were the only oral anticoagulants available for decades.^35^ New oral anticoagulant drugs developed over the past decade target thrombin (IIa) or factor X (Xa) instead of vitamin K. These are known as non-vitamin K inhibiting anticoagulants (NOACs) or direct oral anticoagulants (DOACs). Recent years have seen a rise in the use of NOACs,^15^ which have an improved efficacy-to-safety ratio over VKAs. Additionally, they do not need routine coagulation monitoring and have fewer food and drug interactions compared with VKAs; however, NOACs are more costly and difficult to reverse.^36–38^ Nonetheless, VKAs continue to be commonly prescribed and are the only indicated anticoagulant class for certain indications (eg, antiphospholipid antibody syndrome, mechanical heart valves). With ageing of the population, the number of people with OA and requiring anticoagulation medication will continue to rise. Given our findings of increased risk of OA incidence/progression with VKA use and the lack of effective treatment options for OA, it may be reasonable to consider NOACs over VKAs for medical indications in which NOACs can be used. This may be particularly the case for those who are carriers of the *MGP* risk allele and the *VKORC1* BB-haplotype, which is an estimated 21% of individuals of European ancestry.

The strengths of our study include the robust underlying biological hypothesis, large sample size and high-quality prospectively collected data. However, some limitations of our study should be acknowledged. First, while we found similar results in RS-I and RS-II, analyses in other independent and even larger cohorts are warranted, as in our large sample size, the numbers of acenocoumarol users is still relatively low. Specifically in RSII, which has a much smaller sample size and number of acenocoumarol users compared with RSI. Also replication in non-Central European ancestry-based cohorts is warranted. Second, the association we noted in this study may be due to a shared disease pathology between OA and VKA indications.^39 40^ We addressed this issue by adjusting for multiple cardiovascular disease risk factors in our analysis (hypertension, HDL/total cholesterol ratio, diabetes mellitus, BMI, physical activity, smoking and age). However, we cannot rule out possible confounding by indication. This potential bias needs to be addressed more directly. This could be done by examining direct (new) oral anticoagulants (DOAC/NOAC) users as a comparator group, as these oral anticoagulants do not inhibit the vitamin K cycle. Unfortunately, our study population contains too few DOAC/NOAC users for such an analysis (n=9). Third, as we only have radiographs available of participants whom were able, healthy and survived long enough to come to our research centre at baseline and follow-up visits, our study may contain health and survivor bias. However, this could also possibly indicate that our found effect may even be larger.^20^ Last, as with all observational studies, we cannot rule out residual confounding.

In summary, we found an increased risk of overall OA incidence/progression in users of the VKA acenocoumarol, which was further increased in *VKORC1* BB-haplotype and *MGP* risk allele carriers. These findings are consistent with the known biology of MGP and vitamin K, and are in keeping with prior studies of vitamin K in OA. Taken together, these studies, including the current one, highlight the importance of vitamin K and vitamin K-dependent Gla proteins such as MGP in the pathogenesis of OA. Importantly, our results may also indicate a role for other vitamin K-dependent proteins which occur and function in cartilage and bone tissues, such as osteocalcin and Gla-rich protein.^3^ Given that there are as yet no treatment options for preventing OA onset or progression, vitamin K may represent a modifiable risk factor. These data provide strong rationale for a properly powered randomised clinical trial of vitamin K in an appropriate patient population, such as those with insufficient vitamin K levels and/or MGP risk allele carriers. Additionally, these data lend support to the consideration of DOAC/NOACs in favour of VKAs when appropriate for a supported indication, and highlight the future possibility of genetic screening to identify individuals at high risk of OA incidence/progression.

## Data Availability

Data are available upon reasonable request. All relevant data supporting the key findings of this study are available within the article and its supplementary data. Due to ethical and legal restrictions, individual-level data of the Rotterdam Study (RS) cannot be made publicly available. Data are available upon request to the data manager of the Rotterdam Study Frank van Rooij (f.vanrooij@erasmusmc.nl) and subject to local rules and regulations. This includes submitting a proposal to the management team of RS, where upon approval, analysis needs to be done on a local server with protected access, complying with GDPR regulations.
