# Supplementary material for: Vitamin K antagonist anticoagulant usage is associated with increased incidence and progression of osteoarthritis
Source: Ann Rheum Dis. 2021 Mar 3;80(5):598–604. doi: 10.1136/annrheumdis-2020-219483 (PMC8053344; doi:10.1136/annrheumdis-2020-219483)
Supplement: Supplementary data [file annrheumdis-2020-219483supp001.pdf]

Supplementary Figures

Boer C.G et al., *Vitamin K antagonist anticoagulant usage is associated with increased incidence and progression of osteoarthritis*

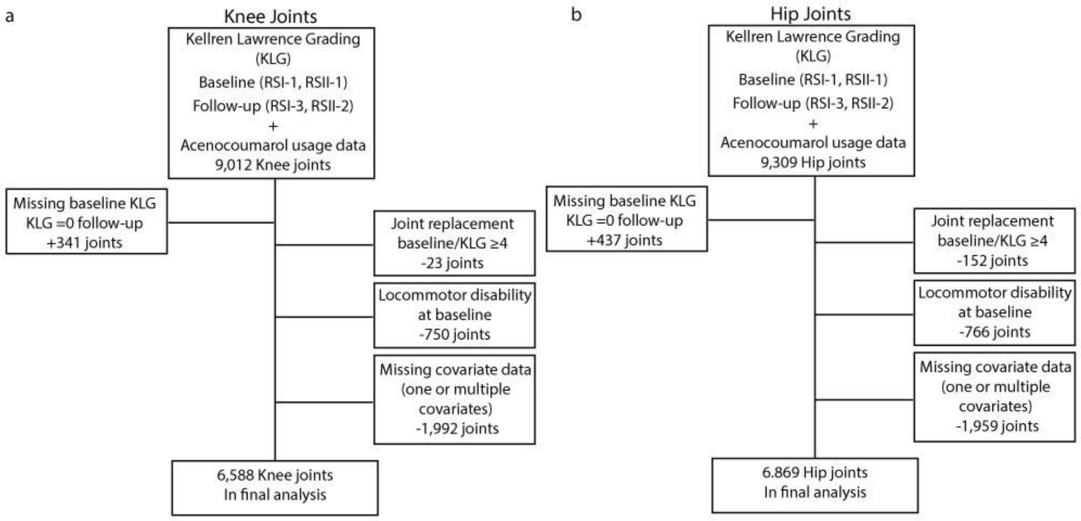

Online Supplementary Figure S1: Flow-chart of included knee and hip joints of the Rotterdam Study a) Flow-chart for the number of knee joints included in the analysis from the Rotterdam study. b) Flow-chart for the number of hip joints included in the analysis from the Rotterdam study. RS: Rotterdam Study, RSI: Rotterdam study subcohort I, RSII: Rotterdam Study subcohort II, KLG: Kellgren-Lawrence Grading.
