# Supplementary material for: Vitamin K antagonist anticoagulant usage is associated with increased incidence and progression of osteoarthritis
Source: Ann Rheum Dis. 2021 Mar 3;80(5):598–604. doi: 10.1136/annrheumdis-2020-219483 (PMC8053344; doi:10.1136/annrheumdis-2020-219483)
Supplement: Supplementary data [file annrheumdis-2020-219483supp002.pdf]

Supplement Appendix for:

Boer C.G et al., *Vitamin K antagonist anticoagulant usage is associated with increased incidence and progression of osteoarthritis*

Contents:

Supplementary Text:

- Computerized pharmacy data of VKA use
- Potential Confounders and risk factors
- Patient and Public Involvement

Supplementary Text

Computerized pharmacy data of VKA use

For 98% of the participants of the RS-I and RS-II, medication usage was available through fully computerized pharmacies within the Ommoord district. All data on dispensed drugs were available in computerized form on a daily basis. Information was available on the date of prescribing, the total amount of drug units for each prescription, the prescribed daily number of units, the product name and the therapeutic chemical code[1]. For each participant, we extracted the usage of VKAs(acenocoumarol) for the period between baseline and follow-up. We extracted information on the prescription of the VKA acenocoumarol. Warfarin is not prescribed, as it is not registered for use as a drug in the Netherlands. All patients taking VKAs in this study attended an anticoagulation clinic, which is standard practice in the Netherlands [2].

Potential Confounders and risk factors

Potential confounders and risk factors were measured at baseline visit (RS-I-1 and RS-II-1). These included age (at baseline visit in years), sex, BMI ($kg^{-1}m^2$), smoking (never, former and current smoker), physical activity (metabolic equivalent of task hours/week), education level (0-3) and lower-limb disability(yes/no) were assessed through home interviews taken by trained interviewers[1]. Education levels were assessed according to UNESCO classification to define education level (0=primary education, 1 = lower/intermediate general education or lower vocational education, 2= intermediate vocational education or higher general education, 3= higher vocational education or university). Locomotor disability was determined for each participant based on the Stanford Health Assessment Questionnaire[3]. Locomotor disability of the lower-limb

was defined as the mean of the scores (with zero indicating no impairment and three indicating unable to perform) on the six questions related to lower limb functions. Disability was defined as a lower limb disability index of 0.5 or over[4]. Femoral neck BMD was measured by DXA-scans as described previously[5]. Arterial hypertension was defined as a systolic blood pressure of 160 mm Hg or higher, diastolic blood pressure of 95 mm Hg or higher, or the use of antihypertensive drugs for hypertension. Diabetes mellitus was defined as use of glucose-lowering medication or non-fasting random or post-load glucose levels exceeding 11.0 mmol/liter. Total cholesterol/HDL ration was determined via serum total cholesterol determined by an enzymatic procedure, HDL measured after precipitation of non-HDL cholesterol. Individuals with missing data on any risk factor and/or confounder were excluded (Supplementary Figure S1).

Patient and Public Involvement

Patients were involved in the design of this study, through the Dutch Arthritis Association (ReumaNederland) through the founding of this research (DAA 2010_017). Patients and the general Public will be informed of the results through the dedicated website of ReumaNederland (https://reumanederland.nl/), and via the Erasmus MC Rotterdam Osteoarthritis Research (ROAR) twitter account (@roar_NL).

References

1. Ikram, M.A., et al., *Objectives, design and main findings until 2020 from the Rotterdam Study.* Eur J Epidemiol, 2020. **35**(5): p. 483-517.
2. Teichert, M., et al., *A genome-wide association study of acenocoumarol maintenance dosage.* Hum Mol Genet, 2009. **18**(19): p. 3758-68.
3. Fries, J.F., P.W. Spitz, and D.Y. Young, *The dimensions of health outcomes: the health assessment questionnaire, disability and pain scales.* J Rheumatol, 1982. **9**(5): p. 789-93.
4. Odding, E., et al., *Association of locomotor complaints and disability in the Rotterdam study.* Ann Rheum Dis, 1995. **54**(9): p. 721-5.
5. Burger, H., et al., *The association between age and bone mineral density in men and women aged 55 years and over: the Rotterdam Study.* Bone Miner, 1994. **25**(1): p. 1-13.
