# Supplementary material for: Vitamin K antagonist anticoagulant usage is associated with increased incidence and progression of osteoarthritis
Source: Ann Rheum Dis. 2021 Mar 3;80(5):598–604. doi: 10.1136/annrheumdis-2020-219483 (PMC8053344; doi:10.1136/annrheumdis-2020-219483)
Supplement: Supplementary data [file annrheumdis-2020-219483supp003.pdf]

Supplement Tables for:

Boer C.G et al., *Vitamin K antagonist anticoagulant usage is associated with increased incidence and progression of osteoarthritis*

Supplementary Table S1. Distribution of *VKORC1* H-Haplotypes in the Rotterdam Study Cohorts

VKORC1 Haplotype	Haplotype Sequence*	RS-I (*n = 12,582*)†	RS-II (*n = 4,314*)†	Total Frequency
Haplotype distribution		*Proportion (number of haplotypes)*		
H1	GGCTAGAGAC	0.13 (1,655)	0.14 (597)	0.13
H2	GGCTCGAGAC	0.24 (3,012)	0.23 (980)	0.24
H3	GGCCAGGGGC	<0.01 (66)	<0.01 (17)	$4.9*10^{-3}$
H4	GGCCAGGCAC	<0.01 (8)	<0.01 (5)	$7.1*10^{-4}$
H5	AGCTCGAGAC	0.02 (221)	0.02 (66)	0.02
H6	AGCCAGGCGC	<0.01 (8)	<0.01 (2)	$5.9*10^{-4}$
H7	AGCCAGGCGT	0.26 (3,238)	0.25 (1,097)	0.26
H8	ATCCAGGCGT	0.12 (1,498)	0.11 (490)	0.12
H9	ATGCAAGCGC	0.22 (2,818)	0.24 (1,022)	0.23
H10‡	GGGCAAGCGC	<0.01 (47)	<0.01 (22)	$4.1*10^{-3}$
Other Haplotypes§	-	<0.01 (11)	<0.01 (17)	$1.7*10^{-3}$
Group distribution				
(Low VKORC1) Group A	H1, H2	0.37 (4,667)	0.37 (1,577)	0.37
(High VKORC1) Group B	H7, H8, H9	0.60 (7,554)	0.60 (2,609)	0.60

* for each haplotype the ten investigated single nucleotide variants (SNVs) are listed in sequential order along the *VKORC1* gene, alleles are coded to the plus-strand, and match the haplotypes reported on PHARMGKB: rs7196161, rs17880887, rs17881535, rs9923231, rs2884737, rs17708472, rs9934438, rs8050894, s2359612 and rs7294.
† Haplotypes have been calculated on all individuals of whom genotype information was available, not only individuals included in our acenocoumarol study population. The total number of haplotypes is twice the number of individuals examined.
‡ The H10 *VKORC1* haplotype has not been described previously[1].
§ Other haplotypes consist of another 16 haplotypes, occurring on average only once in either RS-I or RS-II.
RS: Rotterdam Study, *VKORC1*: Vitamin K Epoxide Reductase Complex 1, H: haplotype

Supplementary Table S2. Association between acenocoumarol use and risk of OA incidence and progression in RSI and RSII

	**Overall Osteoarthritis Progression**					**Overall progression of Knee Osteoarthritis**					**Overall progression of Hip Osteoarthritis**				
	**Joints *N****	**Incidence/ Progression *N* (%)**	**OR**	**95% CI**	**p-value**	**Joints *N****	**Incidence/ Progression *N* (%)**	**OR**	**95% CI**	**p-value**	**Joints *N****	**Incidence/ Progression *N* (%)**	**OR**	**95% CI**	**p-value**
**Non-users**	12,594	506 (4.0%)	1	-	-	6,162	329 (5.3%)	1	-	-	6,432	177 (2.8%)	1	-	-
**Users**	863	94 (10.9%)	2.92	2.32-3.68	< 0.001	426	55 (12.9%)	2.62	1.94-3.56	< 0.001	437	39 (8.9%)	3.46	2.14-4.97	< 0.001
**Duration of Acenocoumarol usage**															
Non users	12,594	506 (4.0%)	1	-	-	6,162	329 (5.3%)	1	-	-	6,432	177 (2.8%)	1	-	-
≤ 180 days	279	35 (12.5%)	3.43	2.38-4.94	< 0.001	144	15 (10.4%)	2.06	1.19-3.56	$9.4*10^{-03}$	135	20 (14.8%)	6.14	3.73-10.11	< 0.001
**>180 days & *≤ 556 days***	285	36 (12.6%)	3.45	2.41-4.95	< 0.001	135	20 (14.8%)	3.08	1.89-5.02	< 0.001	150	16 (10.7%)	4.21	2.46-7.24	< 0.001
>556 days	299	23 (7.7%)	1.99	1.29-3.07	$1.9*10^{-03}$	147	20 (13.6%)	2.79	1.72-4.53	< 0.001	152	3 (2.0%)	0.71	0.22-2.25	0.56

Incidence and progression of osteoarthritis(OA) in RS-I and RS-II within the follow-up time associated with acenocoumarol use. Model used is a GEE (Generalized Estimated Equations) multivariate logistic regression model including acenocoumarol use.

Acenocoumarol usage examined by tertiles: first ≤ *180 days,* second *>180 days and ≤ 556 days, third: >556 days of acenocoumarol use*

*Number of individual knee and/or hip joints studied from RSI and RSII (Supplementary Figure S1 for exclusions)

BMJ Publishing Group Limited (BMJ) disclaims all liability and responsibility arising from any reliance placed on this supplemental material which has been supplied by the author(s)

Supplementary Table S3. Association between acenocoumarol use and risk of overall OA progression in RSI and RSII separately

	**RS-I: Overall progression of Osteoarthritis**					**RS-II: Overall progression of Osteoarthritis**				
**Acenocoumarol usage**	**Joints *N****	**Progression *N* (%)**	**OR adj.**	**95% CI**	**p-value**	**Joints *N****	**Progression *N* (%)**	**OR adj.**	**95% CI**	**p-value**
Non-users	9,178	406 (4.4%)	1	-	-	3,416	100 (2.9%)	1	-	-
Users	745	79(10.6%)	2.39	1.82 - 3.13	< 0.001	118	15 (12.7%)	3.68	1.91 - 7.07	< 0.001
≤ 180 days	244	31 (12.7%)	2.73	1.79 - 4.18	< 0.001	35	4 (11.4%)	4.13	1.35 - 12.59	$1.3x10^{-02}$
>180 days & *≤ 556 days*	237	29 (12.2%)	2.70	1.77 - 4.12	< 0.001	48	7 (14.6%)	5.61	2.21 - 14.25	< 0.001
>556 days	245	19 (7.2%)	1.75	1.05 - 2.89	$3.1x10^{-02}$	35	4 (11.4%)	1.59	0.50 - 5.00	0.43
	**RS-I: Overall progression of Knee Osteoarthritis**					**RS-II: Overall progression of Knee Osteoarthritis**				
Non-users	4.455	256 (5.7%)	1	-	-	1,707	73 (4.6%)	1	-	-
Users	370	47 (12.7%)	2.32	1.64 - 3.30	< 0.001	56	8 (14.3%)	2.61	1.10 - 6.16	< 0.001
≤ 180 days	127	13 (10.2%)	1.67	0.94 - 3.13	$9.8x10^{-02}$	17	2 (11.7%)	2.89	0.59 - 14.12	0.19
>180 days & *≤ 556 days*	113	18 (15.9%)	2.90	1.70 - 1.95	< 0.001	22	2 (7.4%)	2.01	0.41 - 9.86	0.39
>556 days	130	16 (12.3%)	2.57	1.43 - 4.62	$1.6x10^{-03}$	17	4 (23.5%)	2.85	0.81 - 9.97	0.11
	**RS-I: Overall progression of Hip Osteoarthritis**					**RS-II: Overall progression of Hip Osteoarthritis**				
Non-users	4,723	150 (3.2%)	1	-	-	1709	27 (1.6%)	1	-	-
Users	375	32 (8.5%)	2.52	1.62 - 3.92	< 0.001	62	7 (11.3%)	6.52	2.31 - 18.39	< 0.001
≤ 180 days	117	18 (15.4%)	4.69	2.52 - 8.69	< 0.001	18	2 (11.1%)	7.89	1.83 - 33.89	$5.5x10^{-03}$
>180 days & *≤ 556 days*	124	11 (8.9%)	2.72	1.32 - 5.57	$6.3x10^{-03}$	26	5 (19.2%)	1.49	3.68 - 60.4	< 0.001
>556 days	134	3 (2.2%)	0.65	0.20 - 2.13	0.48	18	0 (0.0%)	-	-	-

Overall progression of Osteoarthritis(OA) in RS-I and RS-II within the follow-up time associated with acenocoumarol use. Model used is a GEE (Generalized Estimated Equations) multivariate logistic regression model including acenocoumarol use and adjusted for age, sex, BMI, smoking, time between baseline and follow-up visit, baseline OA severity in Kellgren-Lawrence score, joint modeled, femoral neck BMD, HDL/total cholesterol ratio, physical activity, education level, hypertension and diabetes mellites.

Acenocoumarol usage examined by tertiles: first ≤ *180 days,* second *>180 days and ≤ 556 days, third: >556 days of acenocoumarol use*

OR: odds ratio, CI: confidence interval, Progression: number of joints showing overall progression of either hip or knee joints or both.

*Number of individual knee and/or hip joints studied from RS-I and RS-II.

Supplementary Table S4 : Acenocoumarol usage with OA incidence or OA progression							
**Acenocoumarol usage**	**Joints†**	**Incidence/ Progression**	**OR**	**95% CI**	**OR**	**95% CI**	**P-value**
OA Incidence only					*Adj.*	*Adj.*	*Adj.*
Non-users	11,936	405 (3.4%)	1	-	1	`-	-
Users	792	67 (8.5%)	2.63	2.01-3.44	2.47	1.86-3.27	> 0.001
OA progression only							
Non-users	12,189	101 (0.8%)	1	-	1	-	-
Users	796	26 (3.3%)	4.20	2.73-6.47	2.19	1.11-4.29	$2.3*10^{-02}$

Association of acenocoumarol usage on Incidence or progression of Osteoarthritis(OA) in RSI and RSII. Model used is a GEE (Generalized Estimated Equations) multivariate logistic regression model including acenocoumarol use. Adjusted (Adj.) model is adjusted for age, sex, BMI, smoking, time between baseline and follow-up visit, baseline OA severity in Kellgren-Lawrence score, joint modeled, femoral neck BMD, HDL/total cholesterol ratio, physical activity, education level, hypertension, diabetes mellites and Rotterdam Study Cohort.
OR: odds ratio, CI: confidence interval, Progression: number of joints showing OA progression (from KLG=2, to higher grades or joint replacement), Incidence: number of incidence OA joints (baseline KLG=0 to KLG ≥2).
† Number of individual Knee and Hip Joints included in the analysis.

Supplementary Table S5 : Association between acenocoumarol and VKORC1 haplotypes and MGP genotypes

Acenocoumarol use	Joints†	Incidence/ Progression	OR adj.	95% CI	P-value
**High *VKORC1* haplotype and *MGP* risk allele status**					
Non users *VKORC1* AA/AB and *MGP* A/A	2,745	118 (4.3%)	1	-	-
Non users *VKORC1* AA/AB and *MGP* T/*	4,515	160 (3.5%)	0.85	0.67-1.09	0.19
Non users *VKORC1* BB and *MGP* A/A	1,780	87 (4.9%)	1.15	0.86-1.54	0.38
Non users *VKORC1* BB and *MGP* T/*	2,668	114 (4.3%)	1.01	0.78-1.33	0.89
Users *VKORC1* AA/AB and *MGP* A/A	165	13 (7.8%)	1.72	0.93-3.19	$8.5x10^{-02}$
Users *VKORC1* AA/AB and *MGP* T/*	304	27 (8.9%)	1.96	1.21-3.00	$5.4x10^{-03}$
Users *VKORC1* BB and *MGP* A/A	117	11 (9.4%)	2.35	1.21-4.56	$1.1x10^{-02}$
Users *VKORC1* BB and *MGP* T/*	188	31 (16.5%)	4.18	2.69-6.50	$1.8x10^{-10}$

Overall progression of Osteoarthritis(OA) in RSI and RSII within the follow-up time associated with acenocoumarol use effect *MGP* rs1800801 OA risk variant and *VKORC1* expression/VKA dosage haplotypes. Model used is a GEE (Generalized Estimated Equations) multivariate logistic regression model including acenocoumarol use and adjusted for age, sex, BMI, smoking, time between baseline and follow-up visit, baseline OA severity in Kellgren-Lawrence score, joint modeled, femoral neck BMD, HDL/total cholesterol ratio, physical activity, education level, hypertension, diabetes mellites and Rotterdam Study Cohort. *VKORC1* haplotype groups are based on the H haplotypes, see Supplementary Table S1. For *MGP* risk variants and carriers see Table 1. OR: odds ratio, CI: confidence interval, Progression: number of joints showing overall OA progression, T/*: MGP osteoarthritis risk variant carrier (T/A) or (T/T)

† Number of individual Knee and Hip Joints included in the analysis.
